# A *De Novo* Floral Transcriptome Reveals Clues into *Phalaenopsis* Orchid Flower Development

**DOI:** 10.1371/journal.pone.0123474

**Published:** 2015-05-13

**Authors:** Jian-Zhi Huang, Chih-Peng Lin, Ting-Chi Cheng, Bill Chia-Han Chang, Shu-Yu Cheng, Yi-Wen Chen, Chen-Yu Lee, Shih-Wen Chin, Fure-Chyi Chen

**Affiliations:** 1 Department of Plant Industry, National Pingtung University of Science and Technology, Pingtung 91201, Taiwan; 2 Yourgene Bioscience, Shu-Lin District, New Taipei City 23863, Taiwan; 3 Faculty of Veterinary Science, The University of Melbourne, Parkville, Victoria 3010, Australia; 4 Department of Biotechnology, School of Health Technology, Ming Chuan University, Gui Shan District, Taoyuan 333, Taiwan; Ecole Normale Superieure, FRANCE

## Abstract

*Phalaenopsis* has a zygomorphic floral structure, including three outer tepals, two lateral inner tepals and a highly modified inner median tepal called labellum or lip; however, the regulation of its organ development remains unelucidated. We generated RNA-seq reads with the Illumina platform for floral organs of the *Phalaenopsis* wild-type and peloric mutant with a lip-like petal. A total of 43,552 contigs were obtained after *de novo* assembly. We used differentially expressed gene profiling to compare the transcriptional changes in floral organs for both the wild-type and peloric mutant. Pair-wise comparison of sepals, petals and labellum between peloric mutant and its wild-type revealed 1,838, 758 and 1,147 contigs, respectively, with significant differential expression. *PhAGL6a* (CUFF.17763), *PhAGL6b* (CUFF.17763.1), *PhMADS1* (CUFF.36625.1), *PhMADS4* (CUFF.25909) and *PhMADS5* (CUFF.39479.1) were significantly upregulated in the lip-like petal of the peloric mutant. We used real-time PCR analysis of lip-like petals, lip-like sepals and the big lip of peloric mutants to confirm the five genes’ expression patterns. *PhAGL6a*, *PhAGL6b* and *PhMADS4* were strongly expressed in the labellum and significantly upregulated in lip-like petals and lip-like sepals of peloric-mutant flowers. In addition, *PhAGL6b* was significantly downregulated in the labellum of the big lip mutant, with no change in expression of *PhAGL6a*. We provide a comprehensive transcript profile and functional analysis of *Phalaenopsis* floral organs. *PhAGL6a PhAGL6b*, and *PhMADS4* might play crucial roles in the development of the labellum in *Phalaenopsis*. Our study provides new insights into how the orchid labellum differs and why the petal or sepal converts to a labellum in *Phalaenopsis* floral mutants.

## Introduction

Orchids (Orchidaceae) represent one of the largest families of flowering plants, with more than 25,000 species [1]. Orchid production has become a worldwide important business in the floricultural industry. Potted *Phalaenopsis* is one of the most popular orchids in the trade. The *Phalaenopsis* genus belongs to the Orchidaceae, comprised of approximately 66 species [2], with distribution throughout tropical Asia from Taiwan and Sikkhim, in India, to Australia and the larger islands of the Pacific Ocean [2]. *Phalaenopsis* flowers have a zygomorphic floral structure, including three sepals (in the first floral whorl) and two petals as well as a highly modified inner median tepal called a labellum in the second floral whorl. In addition, *Phalaenopsis* flowers are highly evolved with a gynostemium or column because of the fusion of the male and female reproductive organs [3]. The use of tissue culture technology to massively produce elite *Phalaenopsis* orchid clones has been widely adopted by the orchid industry. However, unpredictable mutations or somaclonal variation may occur during tissue culture. Somaclonal variation, characterized by phenotypic changes of genetic or epigenetic origin [4], has been extensively studied in several plants. Such variation includes morphological traits such as flower color and morphologic features, leaf morphologic features and color, plant height, resistance to disease, improved quality and higher yield [5]. The labellum-like petal of the peloric mutant of *Phalaenopsis* is more common than in other somaclonal variants. Occasionally, a rare sepal peloric mutant has been observed. The orchid peloric mutant is thus valuable for investigating flower development at both morphological and molecular levels.

The genetic and molecular basis of floral organogenesis has been extensively studied in the model species *Arabidopsis thaliana* and *Antirrhinum majus* [6–9] and led to the evolving ABCDE model of five major classes of homeotic selector genes: A, B, C, D and E. Most of these key floral regulatory genes are the MADS-box gene family encoding MIKC-type MADS domain proteins that function as transcription factors (TFs) [10,11] A- and E-class genes control the development of sepals in the first whorl [12]. A-, B- and E-class genes work together to regulate petal formation in the second whorl, whereas B-, C- and E-class genes control stamen development in the third whorl. C- and E-class genes determine carpel development in the fourth whorl. D-class genes are involved in ovule development [7,13,14]. In *Arabidopsis*, the A-class genes include *APETALA1* (*AP1*) and *APETALA2* (*AP2*) [15,16]. The B-class genes are represented by *APETALA3* (*AP3*) and *PISTILLATA* (*PI*) [17,18], the C-class gene *AGAMOUS* (*AG*) [19], the D-class gene *SEEDSTICK*/*AGAMOUS-LIKE11* (*STK*/*AGL11*) [19] and the E-class genes SEPALLATAS (SEP1, SEP2, SEP3, and SEP4) [12,20].

The function of ABCDE model genes appear to be conserved across the angiosperms and provide detailed explanations for their floral morphologic features. However, study of the model has focused primarily on herbaceous plants and has not explained completely how diverse angiosperms evolved. The functions of many other expressed genes during floral development remain obscure [21,22]. The flower of orchid is similar to that of eudicots, with sepals and petals in the first and second whorls. However, during floral initiation in orchid, one of the petals develops into a labellum, which is a distinctive feature of a highly modified floral part for an unusual plant species for the study of flower development [23]. Despite its unique floral morphologic features, the molecular mechanism of floral development in orchid remains largely unclear, and more research is needed to identify genes involved in floral differentiation.

Previous studies of orchid floral development have depicted the involvement of certain MADS box genes, including members of the *AP1*, *AP3/PI*, *DEF/GLO*, *AG*, *AGL6* and *SEP* subfamilies. Orchid A-class genes, such as *ORAP11* and *ORAP13* from *Phalaenopsis* [24], *OMADS10* from *Oncidium* [25] and four MADS genes (*DOMADS2*, *DthyrFL1*, *DthyrFL2* and *DthyrFL3*) from *Dendrobium* [26,27] have been identified. Four B-class *DEF*-like MADS-box genes expressed differently between the wild-type and peloric mutants with lip-like petals in *Phalaenopsis* [28,29]. In addition, *OitaDEF*-like genes exert a key function in the diversification of tepals and lip in *Orchis italica* [30]. A putative floral organ identity gene, *OMADS3* (a paleo*AP3* gene), isolated from *Oncidium* implied it to be an A-function gene regulating floral formation and initiation [31]. In addition, three paleo*AP3* genes, *OMADS5*, *OMADS3* and *OMADS9*, and one *PISTILLATA* gene, *OMADS8*, were characterized in *Oncidium* orchid [32]. In *Habenaria*, three *DEF*-like genes were identified, with *HrGLO1* and *HrGLO2* expressed in sepals, petals and columns but *HrDEF* expression detected only in petals and column [33]. C-class and D-class genes were identified from four orchid species: *Phalaenopsis* (*PhalAG1*, *PhalAG2*) [34], *Dendrobium* (*DthyrAG1*, *DthyrAG2*) [26], *Dendrobium* (*DcOAG1*, *DcOAG2*) [35] and *AGAMOUS*-like genes, denoted *CeMADS1* and *CeMADS2*, from *Cymbidium* [36]. Orchid E-class and *AGL-6* genes were identified from *Oncidium* and *OMADS11* in the *LOFSEP* subclade, *OMADS6* in the *SEP3* subclade, and *OMADS1* and *OMADS7* in the *AGL6* subclade [25,37]. However, this research is in line with current thoughts on how major evolutionary changes in the genetic basis of organ identity were established by gene duplication and the separation of functions. We have a long way to go to fully understand the role of MADS-box genes in orchid evolution.

Recently, next-generation deep-sequencing technology such as Solexa/Illumina RNA-seq and digital gene expression (DGE) have provided new approaches for studying global transcriptome profiling of species that lack reference genome information [38]. RNA-seq is widely used with model and non-model organisms to obtain massive sequence data for molecular marker development, gene discovery and transcriptome profiling [39–44]. So far, transcriptomic analyses have been performed with vegetative and reproductive tissues of *Phalaenopsis* [39,42], *Oncidium* leaf and floral buds [45], *Cymbidium* non-pseudobulb shoot and floral buds [46], *Orchis italica* floral buds [47] and *Ophrys* vegetative and reproductive tissues [48]. However, RNA-seq technology has not been used for transcriptomic analysis of floral-organ development in *Phalaenopsis*.

To better understand floral-organ development of *Phalaenopsis* orchid at the molecular level, we used RNA-seq technology to investigate the expression of a large number of genes, with emphasis on those differentially expressed in floral-organ development of the wild-type and peloric mutant of *Phalaenopsis*. To gain a comprehensive understanding of the *Phalaenopsis* floral development and related processes, we dissected sepal, petal and labellum of 0.2-cm wild-type and peloric mutant flower buds for RNA extraction and detailed transcriptome analysis. Differentially expressed transcripts and their expression patterns were analyzed, and several potential candidate transcripts were found to be regulator factors involved in floral development. We identified genes that are significantly differentially expressed in floral organs by comparing the wild-type and peloric mutant. Our study reveals the functional differentiation and coordination of floral organs and provides insights into possible regulatory networks underlying the development of floral buds in *Phalaenopsis*.

## Materials and Methods

### Plant material

**Fig 1:**
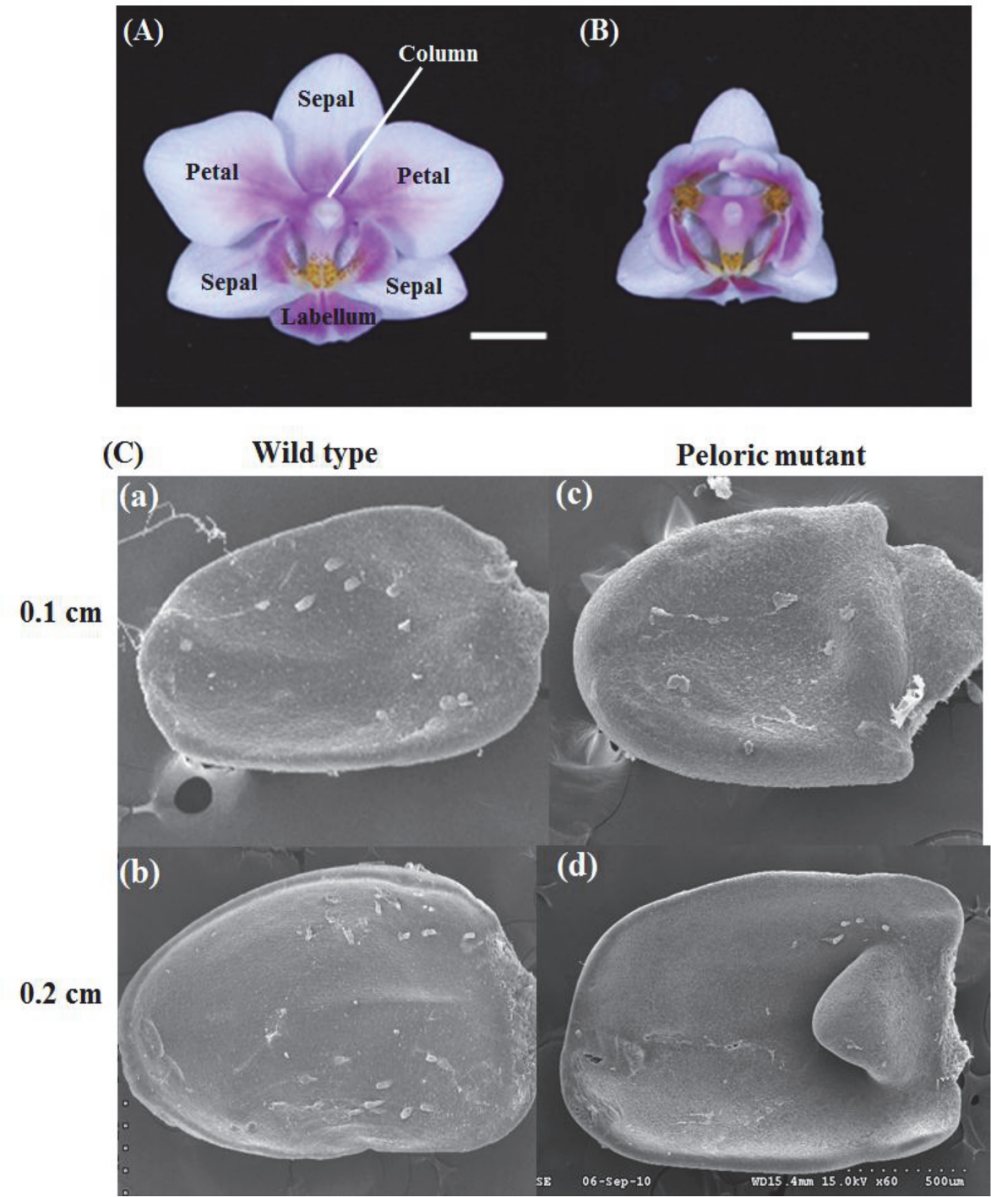
Flowers of wild-type and peloric mutant of *Phalaenopsis* Brother Spring Dancer ‘KHM190’. (A) Wild-type and (B) peloric mutant flower. Bar = 1 cm. (C) Scanning electron microscopy of petal of floral buds at early developmental stages of (a), (b) wild-type and (c), (d) peloric-mutant flower. Bar = 500 μm.

Three distinct floral mutants in petal, sepal or labellum (lip) were used for this study. Wild-type (normal) and peloric petal mutant plants of the orchid hybrid *Phalaenopsis* Brother Spring Dancer ‘KHM190’ (Fig 1 and Fig A in S1 File) were obtained from I-Hsin Biotechnology (Chiayi, Taiwan). Wild-type *Phalaenopsis aphrodite* and its lip-like sepal mutant (peloric sepal) were from own collection. Wild-type flower of *Phalaenopsis* ‘NPU1458’ and its petal-like lip (big lip) mutant was from a breeding population. Wild-type and mutant plants were all grown in fan-and-pad greenhouse of National Pingtung University of Science and Technology (Pingtung, Taiwan) under natural day light and controlled temperature from 27 to 30°C. Flowering plants were maintained in a cooling greenhouse at 20/26°C (night/day) temperature. Virus-infected plants were determined by use of RT-PCR with virus-specific primers of *Cymbidium mosaic virus* (CymMV) and *Odontoglossum ringspot virus* (ORSV) and excluded. For all experiments, sepal, petal and labellum (lip) organs of 0.2-cm flower buds, which were collected from several clone plants derived from tissue culture to provide sufficient source of total RNAs, were collected (Fig A in S1 File), immersed in liquid nitrogen, and stored at—80°C.

### RNA extraction and deep sequencing

RNA was isolated from frozen orchid tissues by the TriSolution method (GeneMark, Taipei) [49]. RNA solution was treated with RNase-free DNase I (Promega, Taipei) to eliminate contaminating DNA. RNA quantity and quality were evaluated by the automated electrophoresis system (Experion, Bio-Rad). RNA samples with RNA quality indicator (RQI) >8 were sent to Yourgene Bioscience on dry ice (New Taipei City, Taiwan) for mRNA purification and cDNA construction. The cDNA library for transcriptome sequencing was constructed with use of the Illumina TruSeq RNA sample prep kit. An amount of 5 μg total RNA was directly fragmented after the oligo-T purification step. The first- and second-strand cDNA was synthesized from the fragmented RNA with random hexamer primers, then underwent end repair, A-tailing, adaptor ligation, size-selection of the range 320–420 bp (approximately insert size 200–300 bp) and then PCR amplification for 15 cycles. The products were loaded onto flow cell channels at 12 pM for pair-end 100 bp×2 sequencing with the Illumina HiSeq 2000 platform (Yourgene Bioscience, New Taipei City, Taiwan).

### *De novo* assembly and analysis of Illumina reads

Before the transcriptome assembly, the clean reads were obtained from raw sequencing reads by removing adaptor sequences, reads with more than 5% unknown nucleotides, and low-quality reads (reads containing more than 50% bases with Q-value ≤ 20). For *de novo* assembly, we pooled all trimmed reads from samples and adopted Velvet (v1.2.07) [50] with distinct k-mer values (35, 45, 55, 65 and 75), followed by Oases (v0.2.06) [51]. The pair-read insert average size was set to 260 bases, with a standard deviation of 10%. Finally, the transcript datasets assembled at different k-mer values were merged by use of Oases with default settings (k-mer = 55).

### Transcriptome annotation

Functional annotation of the contigs involved a local BLASTx search with our assembly against the NCBI Nr database (significant E-value threshold ≤ 1e^-5^). From the search results, to determine gene functions of the sequences, the Blast2GO program was used to obtain gene ontology (GO) annotation according to biological process cellular component and molecular function ontologies [52,53]. Pathway assignments were performed according to the KEGG pathway database [54] with BLASTx and E-value threshold 1e^-5^. Protein domain annotations involved use of RPS-BLAST 2.2.23 against the Pfam database (ver. 25.0) with the best hit and an E-value ≤ 1e^-5^ [55,56].

### Identification of differentially expressed transcripts

To evaluate the expression of raw transcript, we first mapped trimmed reads to raw transcript sequence using gapped alignment mode of the program Bowtie 2.2.1.0 [57]. After alignment, we quantified raw transcript expression with the software package eXpress 1.3.0 [58]. The value of read counts from eXpress would be the input of DESeq [59], an R software package, was used to test for differential expression. Genes with differential expression of at least two-fold change at P ≤ 0.05 between normal and mutant floral organs were identified with the dispersion estimates obtained using blind method in DESeq package.

### Real-time PCR analysis

Quantitative real-time RT-PCR and data analysis involved the ABI PRISM 7300 Sequence Detection System (Applied Biosystems) with SYBR Green PCR Master Mix (Applied Biosystems). Total RNA was isolated from *Phalaenopsis* sepal, petal and lip tissue. To remove contaminating DNA, RNA samples were treated with DNase I and used for first-strand cDNA synthesis by priming with oligo (dT)_25_ and catalyzed with Superscript II Reverse Transcriptase (Invitrogen) at 42°C for 1.5 h. The primers for the transcripts investigated were designed on the basis of open reading frame sequences for each gene with use of Primer Express (Applied Biosystems). The thermal cycling condition was 10 min at 95°C, and 40 cycles of 15 sec at 95°C and 1 min at 60°C. Before running real-time PCR, primer efficiency was evaluated by use of both gene-specific and internal-control *Actin* primers [49,60] (S1 Table) at 50-, 150- and 300-nM combinations as described [49]. We chose the 150-nM concentration for both the target and *Actin* genes as the most suitable combination. Each sample was amplified in triplicate. With the housekeeping gene *Actin*, the relative expression level of target genes was presented as 2^-ΔCT^ by the ΔC_T_ method (Applied Biosystems).

## Results

### *Phalaenopsis* floral morphogenesis in the wild-type and peloric mutant

The *Phalaenopsis* flower features bilateral symmetry with two whorls of tepals and a central gynostemium (Fig 1A). The three petal-like sepals include one at the top and two in the lower lateral positions. The flower has two lateral petals and a specialized, enlarged, flamboyant bottom petal, called a labellum. The gynostemium (column) is a reproductive organ with pistil and stigma organs fused together (Fig 1A). Flowers of peloric mutants are radially symmetrical with lip-like petals due to abnormal protrusion of upper cell layers and may lose their pollinia in severe cases (Fig 1B). Visible differences in the development of wild-type and peloric-mutant floral buds began at stage 2 (0.2-cm bud), with an asymmetric shape of petals. At this stage, the petals in the peloric mutant mimicked the labellum in shape, whereas its sepals were similar to those of the wild-type (Fig A in S1 File). Scanning electron microscopy revealed the stage-2 peloric mutant flower with an abnormal fin-like protrusion on both petals (Fig 1C).

### Assembly of high-quality *Phalaenopsis* flower transcriptomes

To obtain an overview of the *Phalaenopsis* flower transcriptome profiles of stage-2 buds, when the peloric petal began to appear, we used RNA-seq for sequencing six cDNA preparations from sepal, petal and labellum tissues of both the wild-type and peloric mutant and generated 100-bp paired-end reads. Deep-sequencing of the six cDNA samples produced 36,697,424, 58,805,774, 41,084,182, 48,504,500, 78,458,762 and 65,091,470 clean paired-end reads for wild-type and peloric-mutant sepal, petal and labellum transcriptomes, respectively (Table 1). The mean read length was 90 bp. The raw data were submitted to and are available at NCBI and can be accessed in the Short Read Archive (NS: SRX396172; NP: SRX396784; NL: SRX396785; PS: SRX396786; PP: SRX396787; PL: SRX396788). All clean reads were assembled by Velvet and Oases and produced 752,203 assemblies (transcripts). We then chose the one with highest confidence score of a locus or if two isoforms of the same locus have the same confidence score, then the longer one was selected as unigenes. Finally, we obtained 43,552 contigs with a mean length of 1081 nt and a median length of 532 nt. The size distribution of the contigs is in Fig B in S1 File.

**Table 1:** Summary of *Phalaenopsis* floral-organ transcriptome assembly.

Total no. of transcripts	NS	PS	NP	PP	NL	PL
**Raw data**						
Total no. of reads	39,281,522	63,691,838	43,899,068	51,106,016	81,707,498	67,469,926
Total nucleotides (nt)	3,967,433,722	6,432,875,638	4,433,805,868	5,161,707,616	8,252,457,298	6,814,462,526
High-quality reads	36,697,424	58,805,774	41,084,182	48,504,500	78,458,762	65,091,470
**Assembly** *						
Maximum CDS length (bp)	4,816					
Mean CDS length (bp)	1,081					
Total CDS length (bp)	24,915,746					
N50 size (bp)	2,094					
GC percentage	43.36					

NS, wild-type sepal; PS, peloric sepal; NP, wild-type petal; PP, peloric petal; NL, wild-type labellum; PL, peloric labellum (PL)

* All reads mixed.

### Annotation of predicted proteins

**Fig 2:**
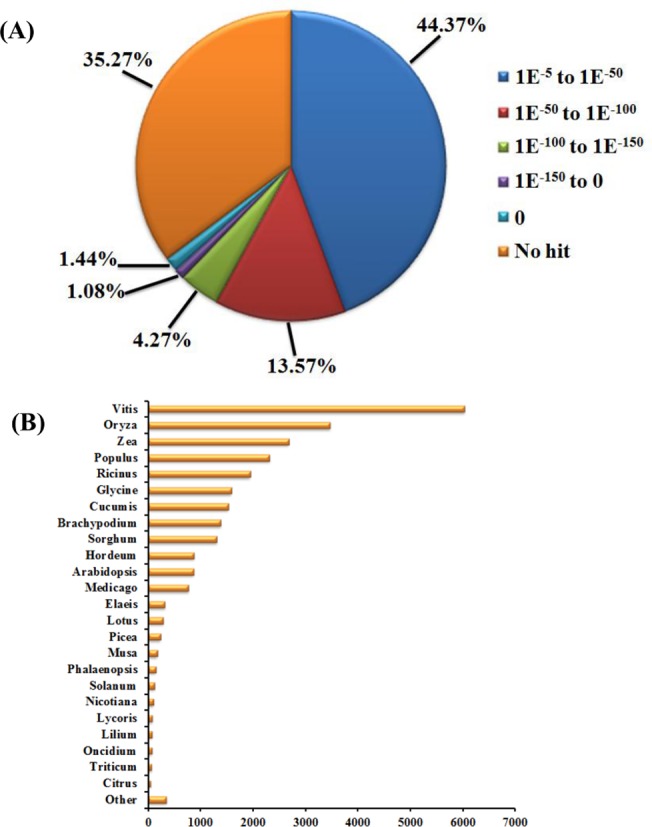
Characterization of sequence homology of the *Phalaenopsis* assembled contigs against Nr databases. (A) E-value distribution of BLASTx hits for the assembled contigs with a cutoff of 1e^-5^ in the NCBI Nr database. (B) Species distribution of the 25 top BLASTx hits shown as number of contigs of the total homologous sequences with an E-value ≥ 1e^-5^.

**Fig 3:**
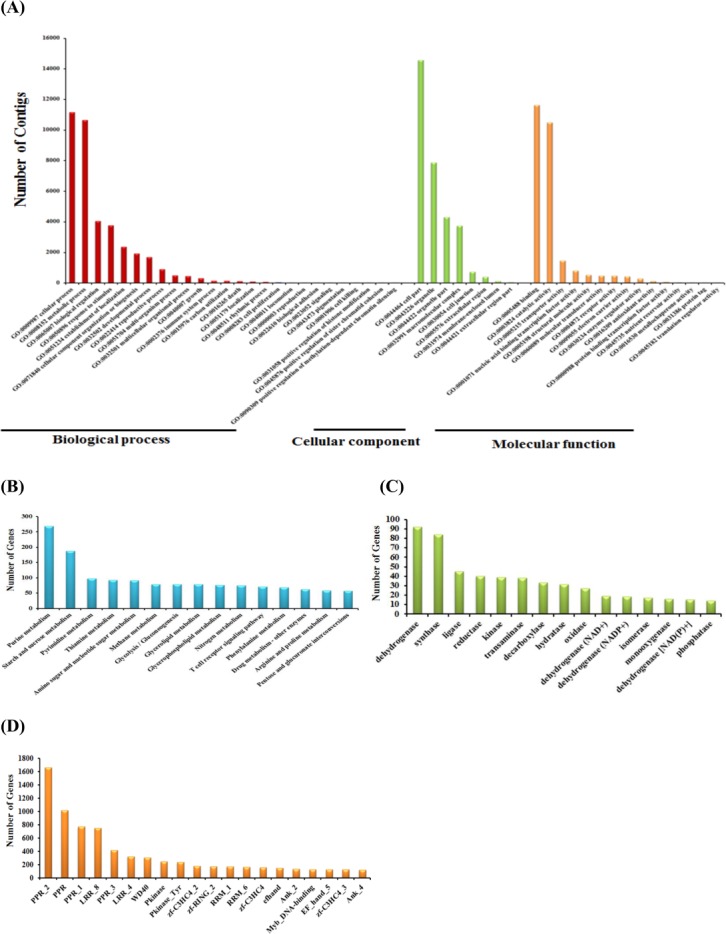
Annotation of the *Phalaenopsis* transcriptome by gene ontology (GO), KEGG and Pfam classification. (A) GO classification summarized by three main categories: biological process, cellular component and molecular function. (B) Functional annotation of transcripts based on KEGG classification. (C) Functional characterization of transcripts for enzyme classes. (D) Pfam domains identified in translated *Phalaenopsis* transcripts.

We annotated, classified, and functionally mapped the 43,552 contig sequences based on BLASTx (cut-off E-value ≤ 1e^-5^) searches of four public protein databases: NCBI non-redundant (Nr) database, gene ontology (GO) database, Kyoto Encyclopedia of Genes and Genomes (KEGG) database, Enzyme Commission (EC) and Pfam (Fig 2; Fig 3 and S2 Table). Among 43,552 contig sequences, significant BLAST hits were found for 28,193 (64.7%) sequences, with no hit found for 15,359 (35.3%) sequences. From the Nr annotations, for the top hits, 44.4% of the annotated sequences (E-value 1e^-5^-1e^-50^) matched available plant sequences (Fig 2A). The species distribution based on Nr annotation is shown in Fig 2B, with the top matches being *Vitis*, *Oryza*, Zea, *Populus* and *Ricinus*.

### Functional annotation and classification of *Phalaenopsis* floral transcriptome

GO assignments were used to classify the functions of the predicted *Phalaenopsis* flower-tissue transcripts. With Nr annotation, we used the Blast2GO 2.3.5 program to obtain GO annotations. In all, 4,609 GO annotations were assigned to 19,712 *Phalaenopsis* flower-tissue contigs, and the terms were summarized into three main GO categories and 49 GO functional groups (Fig 3A). We found 26 subsets within the Biological Process category, 8 within the Cellular Component category and 15 within the Molecular Function category (Fig 3A). Of these, 16,565 (84.0%) comprised the largest category of molecular function, followed by biological process (14,892, 75.6%) and cellular component (14,678, 74.5%) (S3 Table). Thus, the most abundant contigs were related to cellular and metabolic functions in the Biological Process category; cellular component, cell part and organelle functions in the Cellular Component category and molecular function, binding and catalytic activity in the Molecular Function category. Details of the gene annotation for significant hits of the three contig sets are in S3 Table.

To identify biological pathways activated in the flower tissues of *Phalaenopsis*, the assembled contigs were annotated with Enzyme Commission (EC) numbers from BLASTX alignments against the KEGG database (E-value ≤ 1e^-5^). A total of 3,724 transcripts were assigned to 139 KEGG pathways; of these 1,679 transcripts, EC numbers were also assigned (S4 Table). The top 15 KEGG pathways observed for *Phalaenopsis* flower tissue contigs are shown in Fig 3B. A large proportion of such contigs belonged to purine metabolism (map00230), starch and sucrose metabolism (map00500), pyrimidine metabolism (map00240), thiamine metabolism (map00730) and amino sugar and nucleotide sugar metabolism (map00520). The top 15 abundant enzyme classes for *Phalaenopsis* flower tissue contigs were dehydrogenase, synthase, ligase, reductase, kinase and transaminase (Fig 3C and S5 Table).

### Identification of protein-coding domains

We obtained the conserved domain information for the transcriptome in the Pfam database [61] using RPS-BLAST, which scans a set of pre-calculated position-specific scoring matrices with a protein query. Comparison of the 43,552 contigs against the Pfam domain database with E-value cutoff 1e^-5^ and domain coverage >50% resulted in 9,607 contigs matching at least one protein domain model. The most abundant protein domains in *Phalaenopsis* flower buds were the pentatricopeptide repeat (PPR_2, pfam01535; PPR, pfam01535; PPR_1, pfam12854), followed by the leucine-rich repeat (LRR_8, pfam13855), pentatricopeptide repeat (PPR_3, pfam13812), leucine-rich repeat (LRR_4, pfam12799), and WD40 (WD40, pfam00400) domains (Fig 3D and S6 Table).

### Global changes of transcriptome profile in wild-type and peloric-mutant flower organs

**Fig 4:**
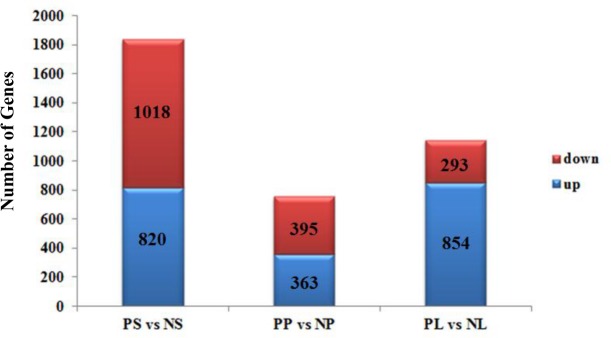
Changes in gene expression profiles between wild-type and peloric-mutant floral organs. The number of up- and downregulated genes in peloric sepal (PS) and wild-type sepal (NS), peloric petal (PP) and wild-type petal (NP), and peloric labellum (PL) and wild-type labellum (NL). Six libraries were summarized.

To identify differentially expressed genes (DEGs) between the wild-type and peloric-mutant flower tissues (sepal, petal and labellum) (Fig 1A and 1B), we determined relative expression levels from our RNA-seq data (43,552 contigs) using DEseq [46]. We found 1,838 distinct contig sequences between the peloric sepal (PS) and wild-type sepal (NS) libraries: 820 were upregulated and 1,018 downregulated in the peloric-mutant sepal (Fig 4). We found 758 distinct contig sequences between the peloric petal (PP) and wild-type petal (NP) libraries: 363 were upregulated and 395 downregulated in the peloric-mutant petal. We found 1,147 distinct contig sequences between the peloric labellum (PL) and wild-type labellum (NL) libraries: 854 were upregulated and 293 downregulated in the peloric-mutant labellum (Fig 4). The expression levels and annotation of these differentially expressed contig sequences are in S7 Table.

### Functional analysis of DEGs based on RNA-seq data

From the functional annotation of the *Phalaenopsis* flower tissue transcriptomes, the GO annotation of DEGs was obtained and underwent GO functional enrichment analysis, specifically the PS vs NS, PP vs NP and PL vs NL comparisons. GO annotation analysis involved groups of genes with greater than two-fold differential expression (log_2_ ratio ≥1 and log_2_ ratio ≤ -1, P< 0.05) in *Phalaenopsis* flower organs. Under PS vs NS, PP vs NP and PL vs NL, the DEGs were classified into 21, 18, and 20 categories of biological processes, respectively, 8 categories each on the basis of cellular components, and 13, 13, and 12 categories on the basis of molecular function (Fig C in S1 File).

### Differential expression of TFs in flower organs

TFs bind DNA and target the assembly of protein complexes to regulate transcript levels of target gene expression [62]. In this study, we performed global TF classification for differentially expressed transcripts and found 878 that were differentially expressed between the wild-type and peloric mutant among *Phalaenopsis* floral organs (sepal, petal and labellum) (S8 Table), including members of the AP2/ERF, ARF, bHLH, homeobox, MADS, MYB and NAC families. This information is valuable in providing a deeper insight into the role of TFs during *Phalalaenopsis* wild-type and peloric-mutant floral-organ development. From DEseq analysis, most TF families from the most abundant transcripts were significantly downregulated and some were upregulated (S8 Table) in the peloric mutant. TFs with previously reported roles in *Phalaenopsis* floral-organ development included *PeMADS4*, which is among the most abundant MADS-box proteins expressed in the *Phalaenopsis* floral organs analyzed. The *PeMADS4* transcript was detected in the wild-type *Phalaenopsis* labellum and ectopically expressed in the petal of the peloric mutant that transformed to a lip-like petal [29]. Another two candidate TFs (CUFF.17763 and CUFF.17763.1), upregulated in lip-like as compared with normal petals, encode a putative TF belonging to the MADS-box gene family that are homologous to *AGL6*-like genes (S8 Table). Some TF families, such as the AP2, homeobox, MYB and NAC family, will be further discussed below. A more in-depth analysis is necessary to determine how these gene families linked to molecular and cellular changes are activated during *Phalaenopsis* floral organ development.

### RNA-seq expression validation by real-time PCR

The Illumina RNA-seq data were validated by real-time PCR analysis of selected genes with RNA isolated from floral organs (sepal, petal and labellum) of the 0.2-cm bud stage of both the wild-type and peloric mutant (Fig 1A and 1B). We selected 27 transcripts with differential expression patterns for real-time PCR analysis and performed a one-by-one comparison of each transcript by real-time PCR and RNA-seq. A total of 21 contigs showed differential expression in agreement with the RNA-seq data (Fig D in S1 File and S9 Table). Three transcripts showing upregulation in the RNA-seq results (CUFF.1482.1, CUFF.40149.1 and CUFF.29848.1) were slightly downregulated in the real-time PCR analysis, one transcript showing downregulation in the RNA-seq results (CUFF.39479.1) was slightly upregulated in the real-time PCR analysis and two transcripts showing downregulation in the RNA-seq results (CUFF. 19890.3 and CUFF. 29789.2) did no show significant difference in the real-time PCR analysis. These two methods yield completely opposite results that genome analyzer provides a holistic picture of all the isoforms of a gene into consideration, whereas the expression by real-time PCR is specific to the isoform of the gene into consideration owing to the use of gene specific primers. Overall, the real-time PCR results agreed well with the RNA-seq data.

### *PhAGL6* may be involved in labellum development of the *Phalaenopsis* flower

**Fig 5:**
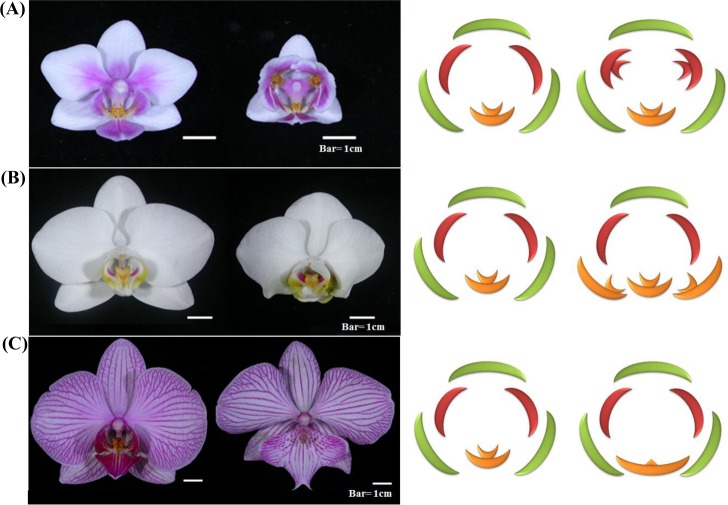
*Phalaenopsis* flower phenotypes of wild-type and peloric mutant. (A) Wild-type flower of *Phalaenopsis* Brother Spring Dancer ‘KHM190’ and its lip-like petal mutant; (B) wild-type flower of *Phalaenopsis aphrodite* and its lip-like sepal mutant; (C) wild-type flower of *Phalaenopsis* ‘NPU1458’ and its big lip mutant. Bar = 1 cm.

**Fig 6:**
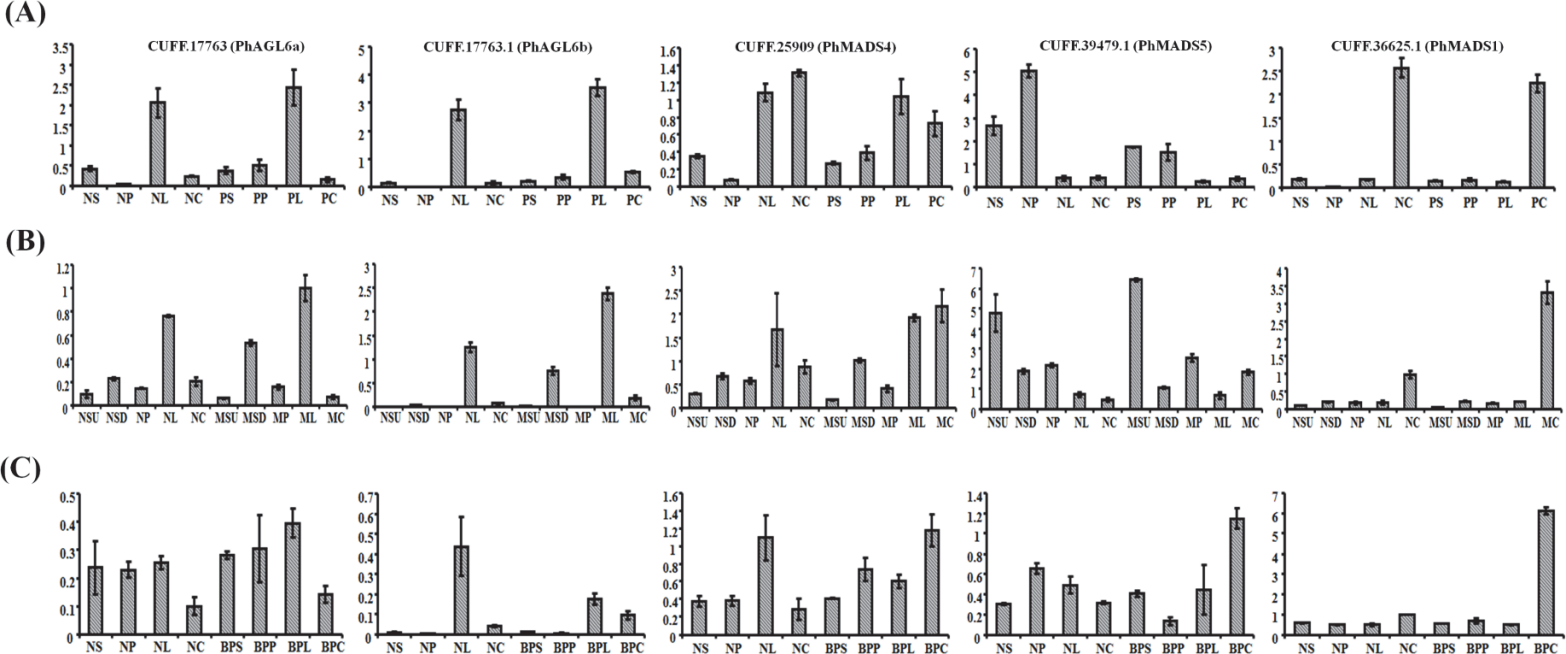
Real-time PCR analysis of genes expressed in different floral mutants of *Phalaenopsis* orchid. Total RNAs isolated from the sepal, petal, lip and column of mature flowers from Fig 5. The wild-type ([A] *Phalaenopsis* Brother Spring Dancer ‘KHM190’; [B] *Phalaenopsis aphrodite* and [C] *Phalaenopsis* ‘NPU1458’) and (N), lip-like petal mutant (P), lip-like sepal mutant (M) and big lip mutant (BP) were used as templates to detect the expression of CUFF.17763(*PhAGL6a*), CUFF.17763.1 (*PhAGL6b*), CUFF.25909 (*PhMADS4*), CUFF.39479.1 (*PhMADS5*) and CUFF.36625.1(*PhMADS1*). S, sepal (include upper and lateral sepals); SU, upper sepal; SD, lateral sepal; P, petal; L, lip; C, column.

To reveal putative roles in labellum development of *Phalaenopsis* flower, we evaluated the association of the expression of five TFs (*PhMADS1*, *PhMADS4*, *PhMADS5*, *PhAGL6a* and *PhAGL6b*) in the floral organs (sepal, petal, labellum and gynostemium) and different floral morphologic features of *Phalaenopsis* orchid mutants (lip-like petal, lip-like sepal, and big lip) from our collection (Fig 5; Fig E in S1 File). We estimated the association of the expression of the five TFs and labellum development by real-time PCR. The *PhMADS1* transcript was highly expressed in the gynostemium (Fig 6). The expression of *PhMADS5* was significantly reduced in lip-like petals of peloric-mutant flowers (Fig 6A) but not in the lip-like sepal of peloric-mutant flowers (Fig 6B). The expression of three TFs (*PhMADS4*, *PhAGL6a* and *PhAGL6b*) was increased in the labellum of the wild-type and mutant (Fig 6). In contrast, *PhMADS4* and *PhAGL6b* transcripts were significantly reduced in the labellum of the big-lip mutant (Fig 6C). Furthermore, the transcript levels of *PhAGL6a* and *PhAGL6b* were significantly increased in the lip-like petal and lip-like sepal of peloric- mutant flowers (Fig 6A and 6B). Thus, *PhMADS4*, *PhAGL6a* and *PhAGL6b* may play different roles in the development of the labellum in *Phalaenopsis*, especially *PhAGL6b* in peloric-mutant flowers and labellum development.

## Discussion

### Construction of an informative floral-organ transcriptome for *Phalaenopsis*

Although the transcriptome of *Phalaenopsis* orchid has been reported previously with expressed sequence tags obtained through Sanger sequencing [49,63], three Roche 454 and Illumina platforms, and three Roche 454 and Illumina sequence datasets available in NCBI [40,43,45], an in-depth comparison of the orchid floral organ transcriptome to reveal developmental cues was lacking. In this study, we used the Illumina HiSeq 2000 sequencing platform to generate six transcriptome sequence datasets of different floral organs (sepal, petal and labellum) from the *Phalaenopsis* wild-type and peloric mutant. The assembled contigs of *Phalaaenopsis* floral organs were further analyzed to generate a functional characterization of the transcriptome and differential expression analysis. We assembled the contigs of the six libraries and obtained 43,552 transcripts; 64.7% of the contigs returned a significant BLAST result. The top five plant species with BLAST hits to annotated contigs were *Vitis*, *Oryza*, *Zea*, *Populus* and *Ricinus* (Fig 2B), for which the annotations of their genomes are comprehensive and largely accepted. Among 43,552 *Phalaenopsis* transcripts, 19,712 had GO annotations, 3,724 mapped to 139 pathways of KEGG, and 9,607 matched at least one protein domain with Pfam (Fig 3A, 3B and 3D). Thus, many unique processes and diverse pathways are involved in *Phalaenopsis* floral organ development.

### Genes commonly expressed in floral organs

Transcript expression profiling is often compared among different developmental stages, different plant organs, or plants under different growth conditions [44,64–67]. In the present study, we identified many genes showing transcriptional changes in the *Phalaenopsis* floral organ by comparing the wild-type and peloric mutant. We identified 1,838 (PS vs. NS, 820 upregulated, 1,018 downregulated), 758 (PP vs. NP, 363 upregulated, 395 downregulated) and 1,147 (PL vs. NL, 854 upregulated, 293 downregulated) DEGs that were differentially expressed by greater than two-fold at P< 0.05 significance (Fig 4 and S7 Table). Most of these genes were involved in regulating flower development, such as floral meristem transformation or establishment of the floral meristem into different types of floral organs [13,68–70]. Our global transcript profiles provide a comprehensive high-resolution analysis of gene expression changes associated with *Phalaenopsis* floral-organ development.

From these DEGs, genes and TFs identified included floral homeotic MADS- box genes, MYB genes and NAC TFs (S8 Table) whose putative functions were linked to the morphogenesis of *Phalaenopsis* flower buds. By using the DEGs datasets, and computational and statistical analyses, this study led to the identification of genes that are likely involved in the control of key developmental processes during *Phalaenopsis* floral-organ development. Further functional characterization of the DEGs by GO functional analysis revealed an additional 42 (PS vs NS), 39 (PP vs NP) and 40 (PL vs NL) categories involved in biochemistry, metabolism, growth and regulation of biological processes (Fig C in S1 File). This DEG information will be valuable to elucidate floral organ development and to find novel floral-organ-related genes specific to *Phalaenopsis* orchids.

### Transcriptional regulation in *Phalaenopsis* floral organs

Regulation of gene expression via TF binding is the primary mechanism by which dynamic complex processes of development and differentiation are controlled [71,72]. The specification of different types of floral organs is a key process regulated by the floral homeotic genes. These genes encode TFs that act in a combinatorial manner to regulate floral-organ developmental programs [6,73,74]. In the present study, we identified 878 TFs using global TF classification for the differentially expressed transcripts. Subsets of TF families were associated with functions in cell differentiation (bZIP, bHLH and MYB), meristem maintenance (homeobox, NAC and YABBY), floral-organ development (MADS and TCP) and other roles in hormone-mediated signalling by auxin (Aux/IAA, ARF), GA (GRAS and Dof) or ethylene (AP2/ ERF). The bHLH family contains genes regulating flower development, such as controlling floral-organ formation as well as the morphogenesis of sepals, petals, stamens and anthers in *Arabidopsis* [75–77], *Eschscholzia californica* [78] and rice [79]. The *Phalaenopsis* bHLH families showed complex expression profiles (S7 Table), which suggests their intricate roles in floral-organ development. Further investigation of bHLH TFs are required to verify floral-development regulation and interaction between these factors and other genes during early labellum and lip-like petal differentiation. The MYB transcription factors have conserved DNA binding domains and some have been known to regulate floral development [80–82]. Most of the *Phalaenopsis* MYB TFs in our RNA-seq data showed marked differential expression among the floral organs examined (S8 Table). Of the four MYB TFs, two (CUFF.26564.1 and CUFF.22705.1) were highly expressed in lip-like petals and the other two (CUFF.15031.1 and CUFF.15465.1) were upregulated in wild-type petals, which suggests different roles played by the four MYBs in petal organ development. The homeobox genes encode a group of transcriptional regulators that control meristem, floral and leaf maintenance and development [83–87]. In our RNA-seq data, one homeobox protein, *KNOTTED-1-like 3* (CUFF.39041.1), was upregulated in lip-like petals. The roles of *KNOTTED-1* genes such as *HIRZINA* and *INVAGINATA* in spur development have been reported in snapdragon (*Antirrhinum majus*) and *Linaria vulgaris* [83,88]. *HIRZINA* and *INVAGINATA* also induced sac-like outgrowths and distally dissected corolla tubes on flowers when constitutively expressed in transgenic tobacco [83]. Of note, *KNOTTED-1-like 3* was ectopically expressed in *Phalaenopsis* lip-like petal, which suggests its probable function in inducing callosity structure and a sawtooth petal formation.

The MADS-box genes encode a family of TFs that are the best-studied floral TF family so far. Members of this family play prominent roles in floral organ specification [11,89]. In our study, the MADS-box TFs showed different expression profiles: four (CUFF.17763, CUFF.17763.1, CUFF.25909 and CUFF.36625.1) were upregulated and one (CUFF.39479.1) was downregulated in lip-like petals of the peloric mutant (S8 Table). In *Phalaenopsis*, *PeMADS4* plays an important role in labellum development because its transcript was restricted to the labellum and lip-like petals of the peloric mutant [29]. Furthermore, the absence of *OMADS5* expression is necessary for the formation of the large lips and the conversion of the sepal or petal into lips in *Oncidium* peloric mutants [32]. In our DEGs and real-time PCR analysis, we identified one transcript encoding *PeMADS4* with higher expression in labellum and lip-like petals (Fig 6A), which is similar to findings by Tsai et al. (2004) [29]. Two members of the *AGL6-like* MADS-box subfamily were upregulated in the lip-like petal of peloric mutants and the labellum (Fig 6A, 6B and 6C). The *AGL6-like* genes define floral organ and meristem identity in *Arabidopsis*, rice, petunia and maize [64,90–93]. The *AGL6-like* genes from our *Phalaenopsis* floral transcriptomes might also play an important role in maintaining labellum development. Ectopic expression of *AGL6-like* genes in transgenic *Phalaenopsis* petals may reveal their function.

### PhAGL6-like and PhMADS4 are critical candidate regulators of labellum development in *Phalaenopsis*

To reveal the regulation of five MADS genes (*PhMADS1*, *PhMADS4*, *PhMADS5*, *PhAGL6a* and *PhAGL6b*) in floral-organ development in *Phalaenopsis*, we examined their expression in different floral morphogens of *Phalaenopsis* orchid mutants (lip-like petal, lip-like sepal and big lip). The increased *PhMADS4* transcript level in labellum and gynostemium of wild-type plants suggests its positive role in both labellum and gynostemium formation (Fig 6A, 6B and 6C). The *PhMADS4* transcript level was 5.05-fold higher in lip-like petals of peloric mutant flowers than in wild-type petals (Fig 6A). In addition, the *PhMADS4* transcript was also 1.48-fold increased in mutant lip-like sepal organ and 0.55-fold decreased in big-lip organs (Fig 6B and 6C). The results from these two different flower mutants suggest that *PhMADS4* might be involved in labellum development and lip-like petal formation, as reported by Tsai et al. (2004) [29]. However, in the lip-like sepal of peloric mutants, *PhMADS4* might play only a minor role in the conversion of the sepal into a labellum. The increased *PhMADS4* transcript may be a secondary effect of sepal conversion into a lip-like structure. This assumption still requires further investigation. The expression of *PhMADS5* was upregulated in sepals and petals but upregulated only in gynostemium of the big-lip mutant. *PhMADS5* transcript level was slightly but significantly decreased (0.297-fold) in lip-like petals and decreased (0.56-fold) in lip-like sepals. This finding implies that *PhMADS5* may be related to petal and sepal development. In all wild-types and peloric mutants examined, *PhMADS1* transcript expression was stronger in the gynostemium than sepal, petal and labellum (Fig 6A, 6B and 6C), as reported by Chen et al. (2012) [94]. *PhMADS1* is a C-function homeotic gene, which is associated with the formation of gynostemiums [94].

**Fig 7:**
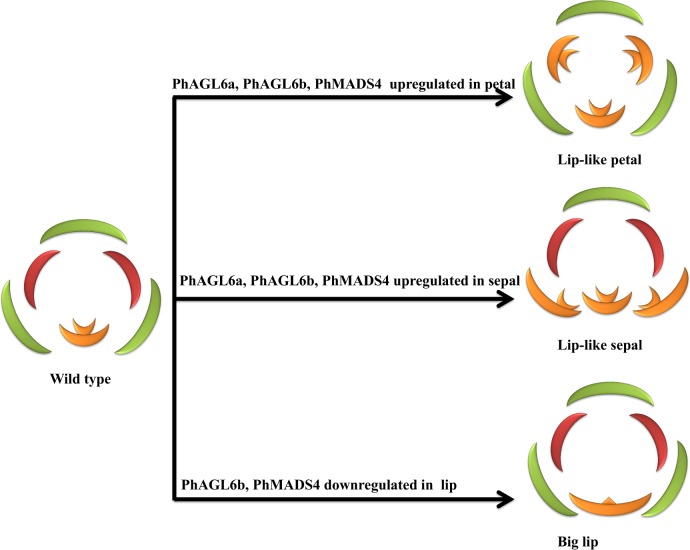
Possible evolutionary relationships between *PhAGL6a*, *PhAGL6b* and *PhMADS4* in the regulation of lip formation in *Phalaenopsis* orchid.

The expression of two *AGL6-like* TFs (*PhAGL6a* and *PhAGL6b*) was increased in the labellum of both the wild-type and lip-like petal mutant (Fig 6A). Furthermore, the expression of *PhAGL6a* and *PhAGL6b* was significantly increased in the lip-like petal of peloric mutant flowers. *AGL6-like* genes may be a positive regulator of labellum formation. Ectopic expression *AGL6-like* genes in petal or sepal may convert them into a lip-like structure. In contrast, downregulation of *AGL6-like* genes in the labellum may affect labellum development. As a further test of the significance of these assumptions, we examined the expression patterns of *PhAGL6a* and *PhAGL6b* in the lip-like petal, lip-like sepal and big lip of peloric mutant flowers. As expected, *PhAGL6a* and *PhAGL6b* were ectopically expressed in the lip-like petal and lip-like sepal organs in peloric mutants. In addition, the expression of *PhAGL6b* was reduced in the labellum of the big-lip mutant, with no change in expression of *PhAGL6a*. In summary, *PhMADS4*, *PhAGL6a* and *PhAGL6b* together play different roles in maintenance of labellum development. For petal and sepal mutants converted into a lip-like structure, *PhMADS4*, *PhAGL6a* and *PhAGL6b* are ectopically expressed in the peloric petal and sepal (Fig 7). Alternatively, *PhMADS4* and *PhAGL6b* may be critical and involved in the labellum converting into a big lip (Fig 7).

## Conclusions

We examined global transcriptome landscapes from six floral organ tissues of the wild-type and peloric-mutant *Phalaenopsis* orchid and identified preferentially expressed genes within and among floral organ developmental tissues. By comparing genes showing floral-organ-preferential expression patterns between the wild-type and peloric mutant, we identified DEGs across a wide range of transcript abundances. Cumulative counts of contigs that mapped to predicted gene models enabled the identification of functionally interesting genes and gene families with altered expression in peloric mutants. Functional analysis and characterization of the differentially expressed genes provides new insight into peloric floral morphogenesis, in particular regarding the role of regulation for floral organ development. We provide a comprehensive list of genes that might be involved in labellum formation and petal conversion into a labellum process. Many TF genes, especially MADS-box genes were expressed in floral organs. We identified the MADS-box genes *PhAGL6a*, *PhAGL6b* and *PhMADS4* as potential regulatory components of labellum development based on conversion of the petal or sepal mutant into a lip-like structure. Our study gives new insights into exploring *PhAGL6a*, *PhAGL6b* and *PhMADS4* transgenic plants as a tool to investigate labellum development and petal or sepal conversion into a labellum in *Phalaenopsis* orchids. Our results provide a strong basis for future research into floral organ development in *Phalaenopsis* orchid.

## Supporting Information

S1 File Supporting figures. (DOC)

S1 Table Real-time PCR primers used in this work. (XLSX)

S2 Table Overview of *Phalaenopsis* floral-organ transcriptome sequencing and assembly. (XLSX)

S3 Table GO categories assigned with *Phalaenopsis* floral-organ contigs. (XLSX)

S4 Table KEGG pathway categories assigned with *Phalaenopsis* floral-organ contigs. (XLSX)

S5 Table EC number categories assigned with *Phalaenopsis* floral-organ contigs. (XLSX)

S6 Table Pfam domains in *Phalaenopsis* floral-organ contigs. (XLSX)

S7 Table Transcripts differentially expressed between peloric-mutant and wild-type flowers. (XLSX)

S8 Table Summary of the annotated transcription factors. (XLSX)

S9 Table DEseq of selected genes in peloric-mutant and wild-type flower. (XLSX)
