# Supplementary material for: A De Novo Floral Transcriptome Reveals Clues into Phalaenopsis Orchid Flower Development
Source: PLoS One. 2015 May 13;10(5):e0123474. doi: 10.1371/journal.pone.0123474 (PMC4430480; doi:10.1371/journal.pone.0123474)
Supplement: S1 File — (DOC) [file pone.0123474.s001.doc]

**Supplementary Figure Legend**

**Fig. A** **in S1 File.** (A) Floral parts of *Phalaenopsis* Brother Spring Dancer ‘KHM190’ wild-type at different stages. A <0.2 cm, B 0.2 cm, C 0.3 cm, D 0.4 cm, E 0.5 cm, F 0.6 cm, G 0.7 cm, H 0.8 cm, I 0.9 cm, J >1 cm, K fully-open flower. (Bar= 2 mm)

(B) Floral parts of *Phalaenopsis* Brother Spring Dancer ‘KHM190’ peloric mutant at different stages. A <0.2 cm, B 0.2 cm, C 0.3 cm, D 0.4 cm, E 0.5 cm, F 0.6 cm, G 0.7 cm, H 0.8 cm, I 0.9 cm, J >1 cm, K fully-open flower. (Bar= 2 mm)

**Fig. B in S1 File.** Contig length distribution of the *Phalaenopsis* floral-organ transcriptome.

**Fig. C in S1 File.** Functional categorization of differentially expressed genes between wild-type and peloric floral organs.

(A) Between peloric sepal (PS) and wild-type sepal (NS).

(B) Between peloric petal (PP) and wild-type petal (NP).

(C) Between peloric labellum (PL) and wild-type labellum (NL).

**Fig. D in S1 File.** Quantitative PCR (qPCR) validation of the relative expression levels of transcripts selected from the differentially expressed gene analysis.

(A) Peloric sepal (PS) vs. wild-type sepal (NS)

(B) Peloric petal (PP) vs. wild-type petal (NP)

(C) Peloric labellum (PL) vs. wild-type labellum (NL)

Expression profiles of selected genes as determined by real-time PCR (line) and differentially expressed genes (box). The signal intensity of each transcript was normalized to that of *Actin*. Data are mean±SD (n = 3)

**Fig. E in S1 File.** Floral parts of *Phalaenopsis* Brother Spring Dancer ‘KHM190’ and *Phalaenopsis* *aphrodite*

(A) Wild-type flower of *Phalaenopsis* Brother Spring Dancer ‘KHM190’

(B) Lip-like petal mutant of *Phalaenopsis* Brother Spring Dancer ‘KHM190’

(C) Wild-type flower of *Phalaenopsis* *aphrodite*

(D) Lip-like sepal mutant flower of *Phalaenopsis* *aphrodite*

(E) Lip-like sepal organs of *Phalaenopsis* *aphrodite*

**A**


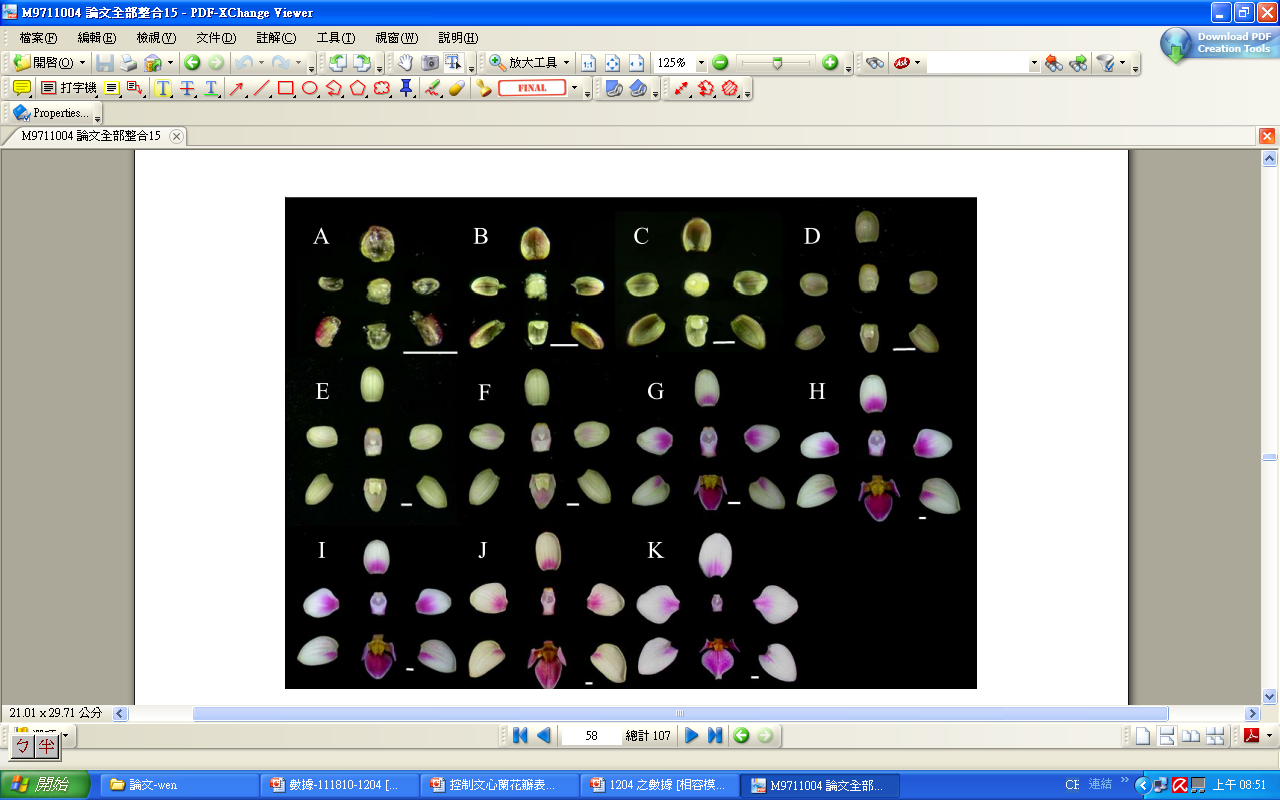


**B**


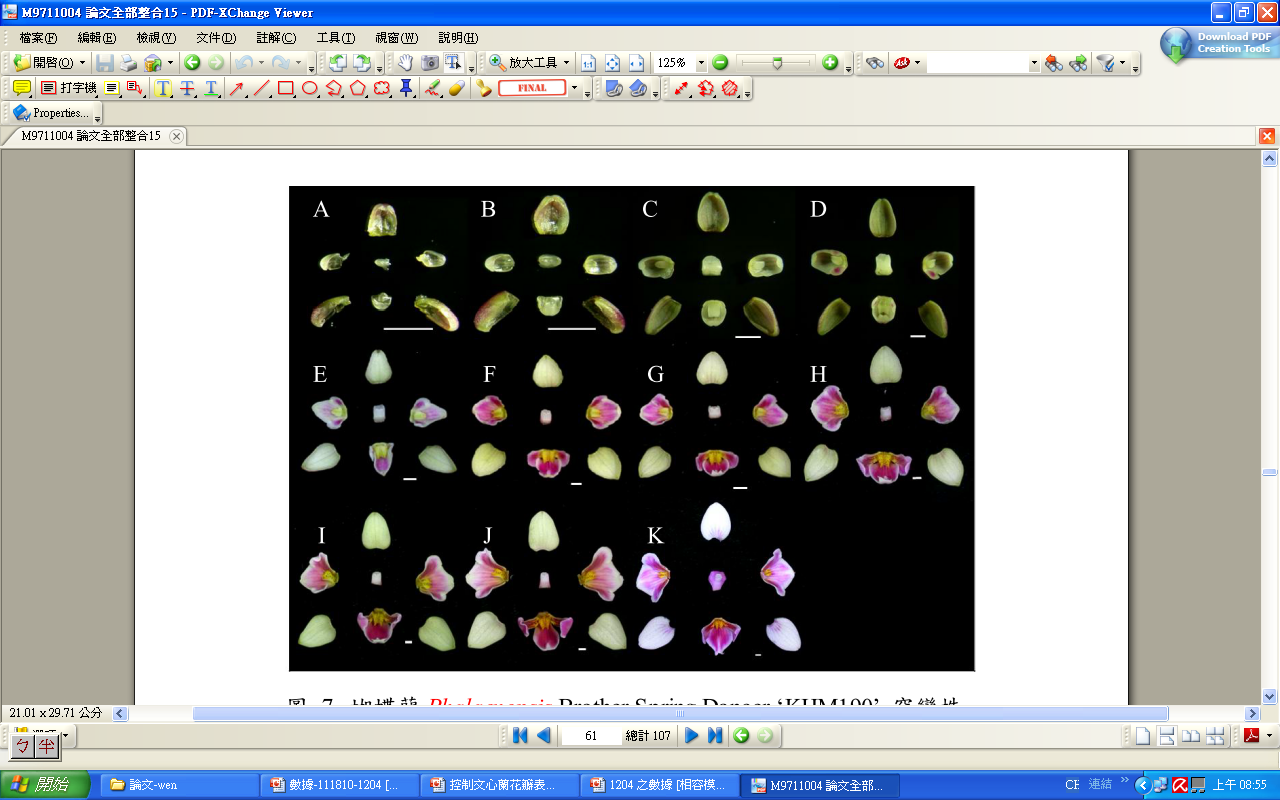


**Fig. A in S1 File.**

**
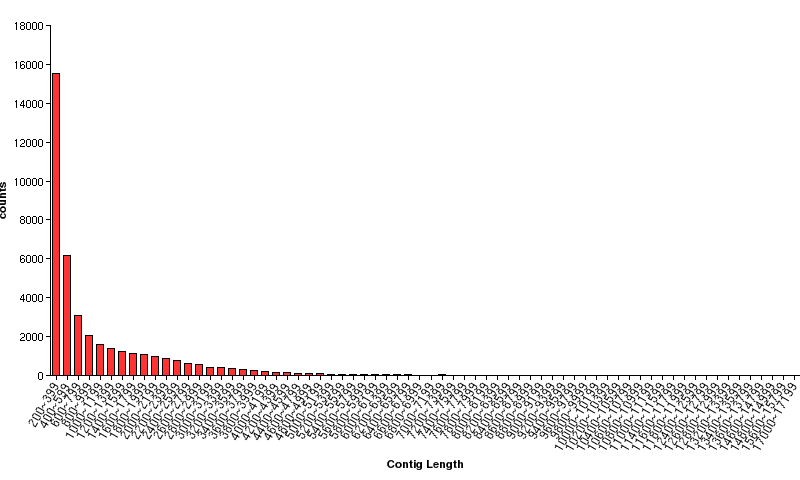
**

**Fig. B in S1 File.**

**A**

**
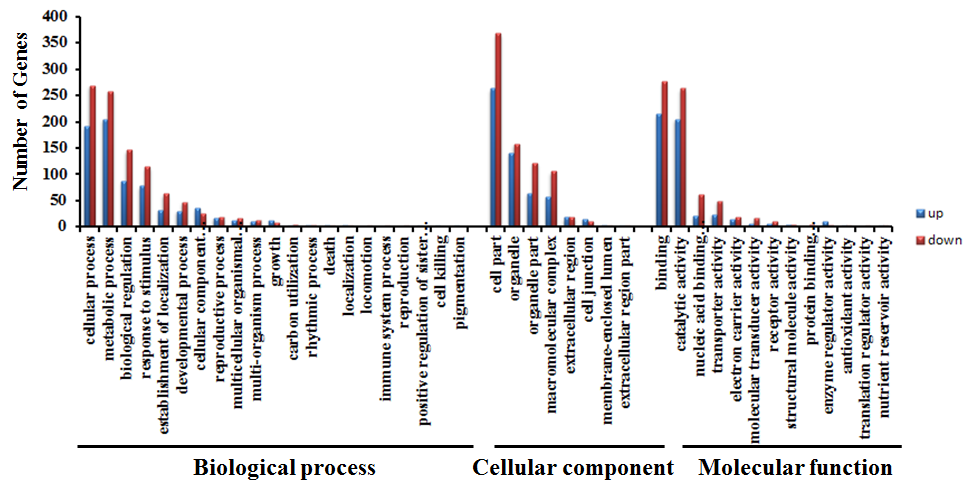
**

**B**

**
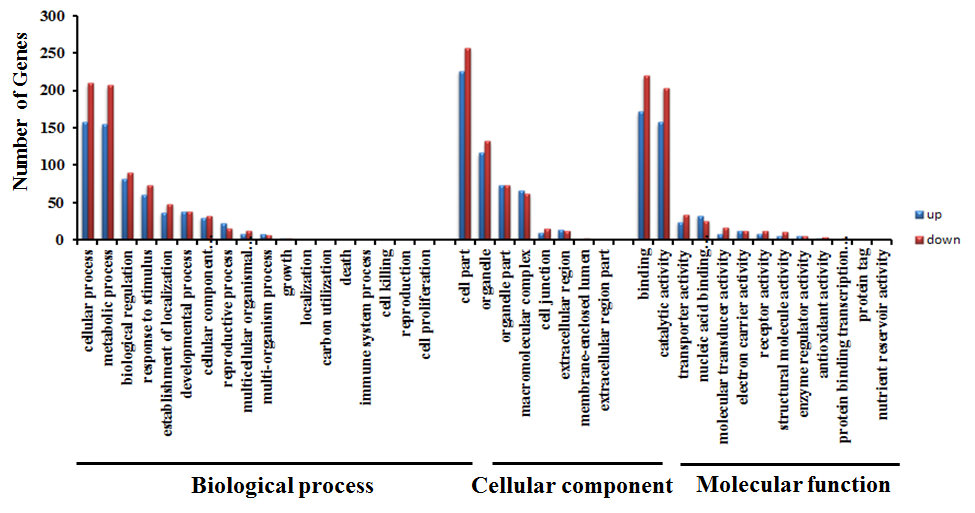
**

**C**

**
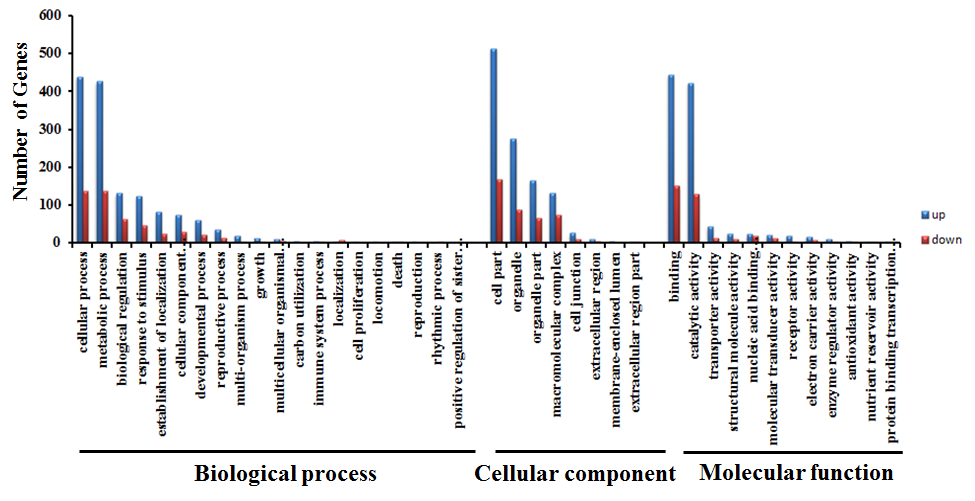
**

**Fig. C in S1 File.**

**A
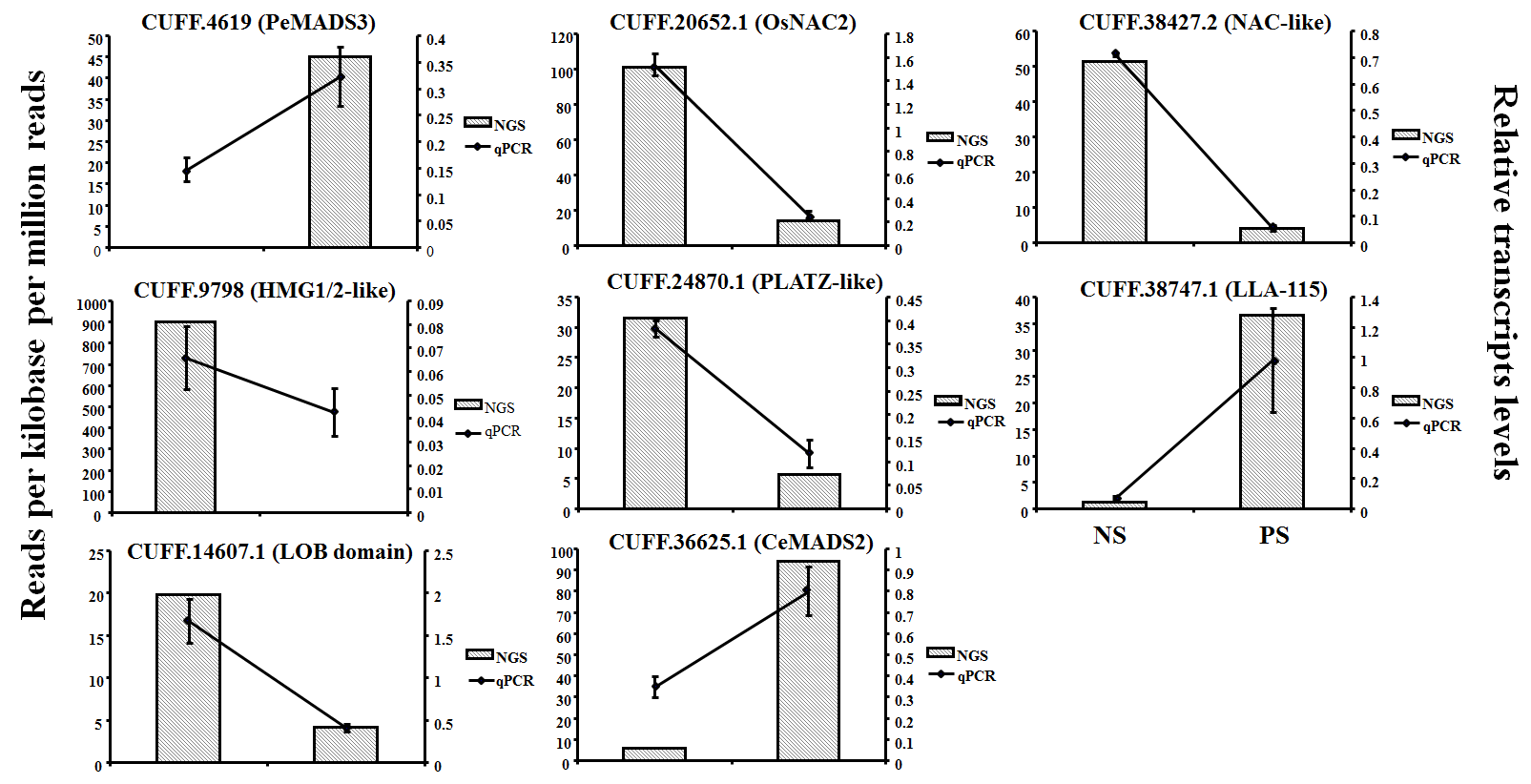
**

**B**

**
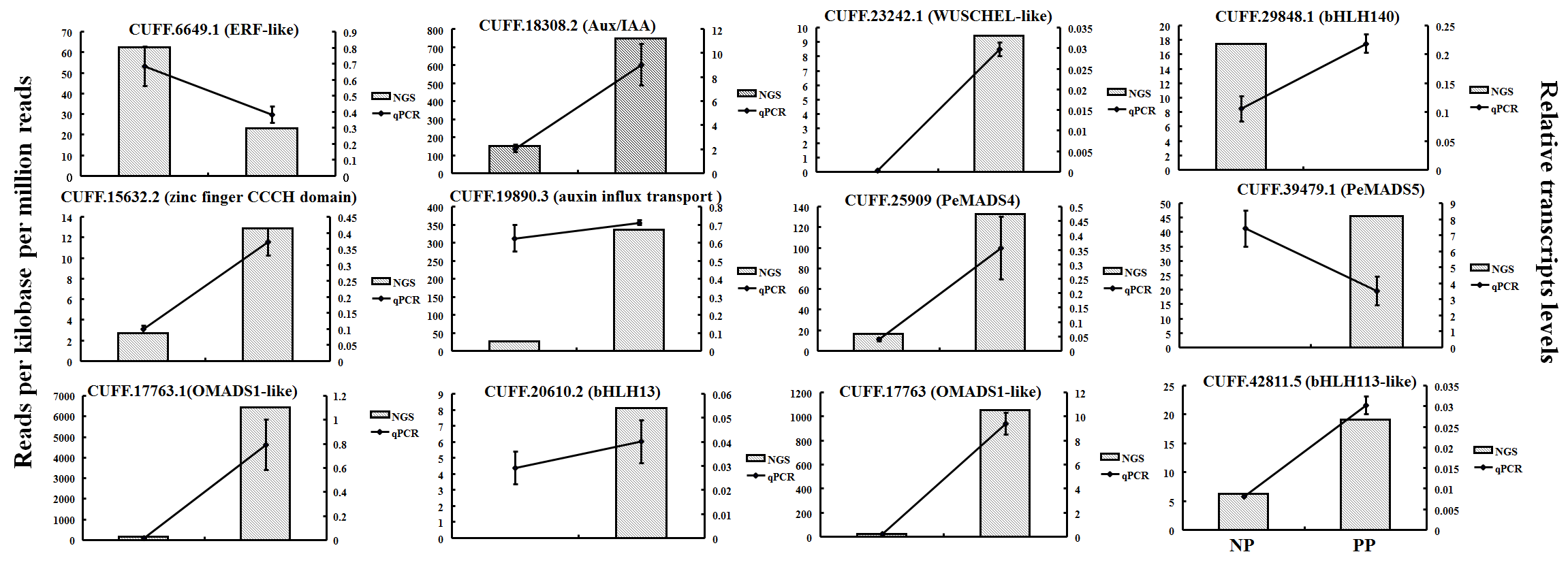
**

**C**

**
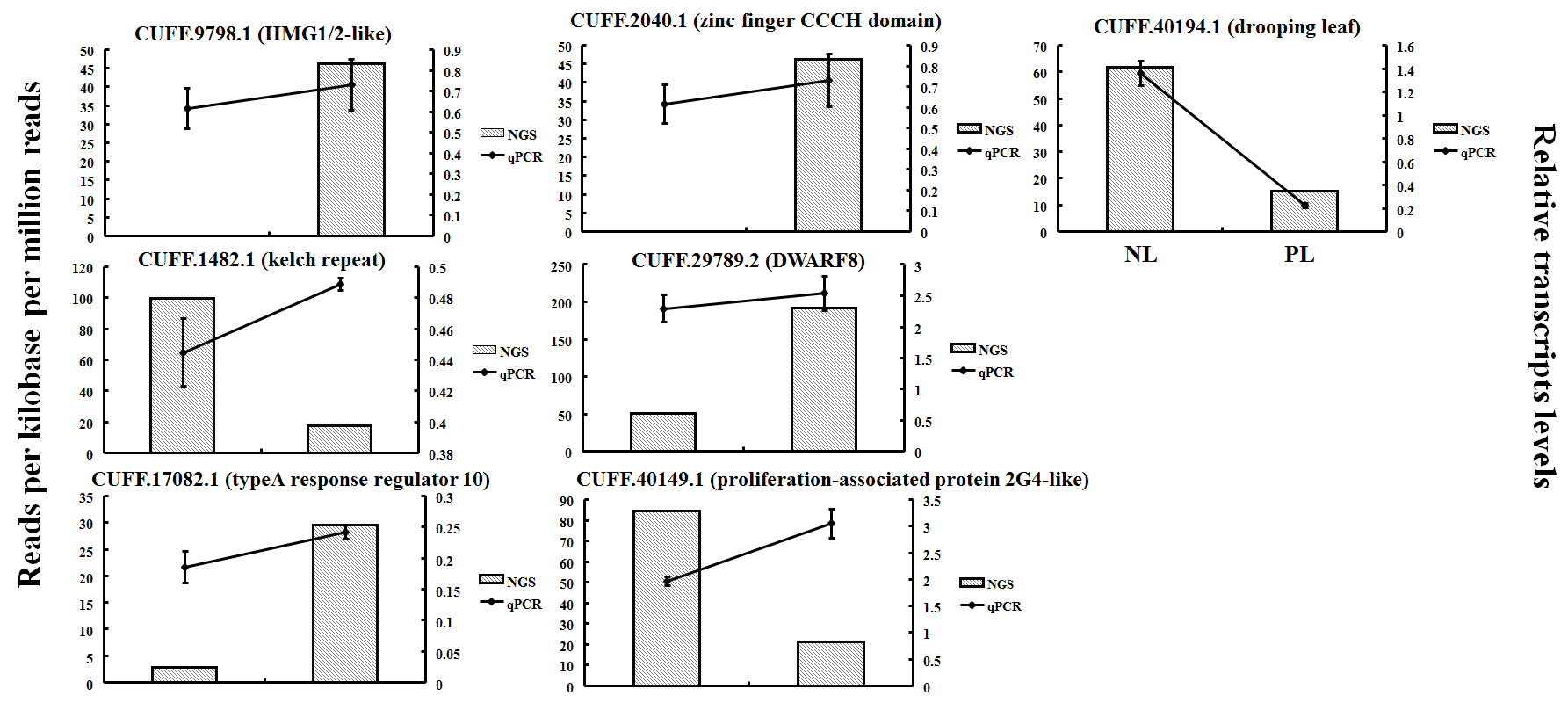
 Fig. D in S1 File.**

**
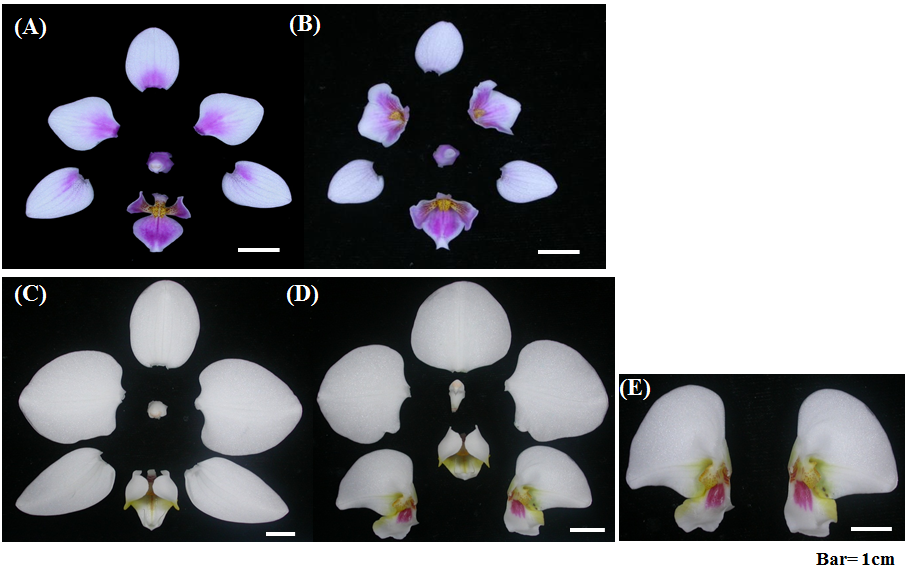
**

**Fig. E in S1 File.**
